# Concurrent Diagnosis of Adenomyosis and Congenital Uterine Anomalies: A Review

**DOI:** 10.3390/jpm13050716

**Published:** 2023-04-24

**Authors:** Edwin Feghali, Andrea Etrusco, Joe Haydamous, Amal Ayed, Antonio Simone Laganà, Vito Chiantera, Salvatore Giovanni Vitale, Stefano Angioni, Guglielmo Stabile, Zaki Sleiman

**Affiliations:** 1 Department of Obstetrics and Gynecology, Lebanese American University Medical Center, Beirut 1100, Lebanon; edwin.feghali@lau.edu; 2 Unit of Gynecologic Oncology, ARNAS “Civico—Di Cristina—Benfratelli”, Department of Health Promotion, Mother and Child Care, Internal Medicine and Medical Specialties (PROMISE), University of Palermo, 90127 Palermo, Italy; etruscoandrea@gmail.com (A.E.); antoniosimone.lagana@unipa.it (A.S.L.); vito.chiantera@unipa.it (V.C.); 3 Department of Obstetrics and Gynecology, University of Balamand, Beirut 1100, Lebanon; joehaydamous@outlook.com; 4 Department of Obstetrics and Gynecology, Farwaniah Hospital, Ministry of Health, Kuwait City 085700, Kuwait; ayedamal@gmail.com; 5 Division of Gynecology and Obstetrics, Department of Surgical Sciences, University of Cagliari, 09124 Cagliari, Italy; salvatoreg.vitale@unica.it (S.G.V.); sangioni@yahoo.it (S.A.); 6 Department of Obstetrics and Gynecology, Institute for Maternal and Child Health-IRCCS “Burlo Garofolo”, 34137 Trieste, Italy; guglielmost@gmail.com

**Keywords:** adenomyosis, congenital uterine anomalies, pelvic pain

## Abstract

**Background:** Adenomyosis and congenital uterine anomalies (CUAs) can compromise reproductive potential and may coexist in the same patient, especially in cases of infertility. This review (CRD42022382850) aims to evaluate the published cases of concurrent adenomyosis and syndromic and nonsyndromic CUAs. **Methods:** A literature search for suitable articles published in the English language was performed using the following databases from inception to 30 November 2022: MEDLINE, EMBASE, Global Health, the Cochrane Library, Health Technology Assessment Database, and Web of Science. Articles including both CUAs and adenomyosis, with data about their potential relationship, were included. **Results:** The literature search retrieved 14 articles that met the purpose of this review and summarized the most recent findings regarding the concurrent diagnosis of adenomyosis and CUAs. **Conclusions:** Adenomyosis can be found in both syndromic and nonsyndromic CUAs, and may arise from several etiologies. The hypothesis that obstructions in CUAs increase uterine pressure and promote the development of adenomyosis remains to be further elucidated, and additional findings may also play a role. The patient’s genetic, epigenetic, and hormonal patterns, as well as normal physiological processes, such as pregnancy, may influence the growth of adenomyosis.

## 1. Introduction

Congenital uterine anomalies (CUA) include a wide diversity of uterine morphologies that might compromise reproductive potential. They are caused by the embryological altered development of the paramesonephric or Müllerian ducts and are linked to decreased fertility and a higher risk of unfavorable reproductive and maternal–fetal outcomes [1]. The European Society of Human Reproduction and Embryology (ESHRE) and the European Society for Gynecological Endoscopy (ESGE) developed a new updated anatomy-based classification system for CUAs, classifying them into six different categories [2]. In complex malformation, the definition of the exact category requires both hysteroscopy and laparoscopy [3]. Furthermore, CUAs can be concurrent with several gynecological diseases, teratogenic interference, and complex genetic syndromes [4]. Indeed, their correlation with endometriosis has been frequently reported [5], and because both endometriosis and CUAs are known to affect conception [6], they are frequently found during infertility work-ups; in particular, endometriosis is sometimes concurrent with obstructive and nonobstructive uterine abnormalities, such as septate uteri [7]. Nevertheless, data about the concurrent diagnosis of adenomyosis and CUAs are still elusive. Adenomyosis is often diagnosed in women of reproductive age, mainly in those with menorrhagia and dysmenorrhea, abnormal uterine bleeding (AUB), dyspareunia, or infertility, although one third of them are asymptomatic [8]. The etiology and pathogenesis of adenomyosis remain to be elucidated, although recent advances in diagnostic techniques and molecular insights have paved the way for a better understanding of this condition [9,10]. For instance, a pathogenic theory of adenomyosis suggests that the invagination of the basalis endometrium into the myometrium is caused by an altered or interrupted junctional zone (JZ), which is a highly specialized hormone-responsive structure found in the inner third of the myometrium [11]. Moreover, chronic peristaltic myometrial contractions may cause continual microtrauma to the JZ, resulting in inflammation and localized increased estrogen production, creating a vicious positive feedback loop. Thus, tissue damage to the endometrial–myometrial interface increases the risk of adenomyosis and supports the concurrent diagnosis of the latter with previous cesarean section, multiparity, or uterine surgery [8]. A different pathogenic explanation for adenomyosis suggests that it develops from embryonic or adult stem cell metaplasia in the myometrium; according to this theory, adenomyotic foci are formed when intramyometrial embryonic pluripotent Müllerian remnants undergo metaplastic alterations in the postpubertal uterine wall, resulting in the formation of de novo ectopic endometrial tissue in the context of the myometrial wall [12,13]. Despite all these potential causes, the concurrent diagnosis of adenomyosis and CUAs is scarcely reported in the literature. Considering this element, we performed a review to evaluate the published cases of concurrent adenomyosis and syndromic and nonsyndromic CUAs.

## 2. Materials and Methods

A review was undertaken through a search of the following databases: MEDLINE, EMBASE, Global Health, the Cochrane Library, Health Technology Assessment Database, and Web of Science, and research registers. The review was registered in PROSPERO (CRD42022382850) before starting the search and followed the Preferred Reporting Items for Systematic Reviews and Meta-Analyses (PRISMA) guidelines [14], validated by the Enhancing the Quality and Transparency of Health Research (EQUATOR) network and the *Cochrane Handbook* [15].

We used the medical subject heading (MeSH) term “Adenomyosis” (MeSH unique ID: D062788) in combination with “Congenital” (MeSH unique ID: Q000151), “Uterine Anomalies” (MeSH unique ID: C562565), “Congenital Abnormalities” (MeSH unique ID: D000013), “Uterine Duplication Anomalies” (MeSH unique ID: D000093662), “Uterine Didelphys” (MeSH unique ID: D000093642), “Bicornuate Uterus” (MeSH unique ID: D000093663), and “Septate Uterus” (MeSH unique ID: D000093665). We selected papers written in English from the inception of each database until 30 November 2022.

Titles and/or abstracts of studies retrieved using the search strategy and those from additional sources were screened independently by 2 review authors (A.E. and J.H.) to identify studies that potentially meet the aims of the review. The full texts of these potentially eligible articles were retrieved and independently assessed for eligibility by other 2 review team members (A.A and A.S.L.). Any disagreement between them over the eligibility of particular articles was resolved through discussion with a third (external) collaborator. We selected only cohort (retrospective and prospective), clinical, or case-control studies, case reports, case series, review articles, theoretical articles, and retrospective reviews reporting the concurrent diagnosis of adenomyosis and CUAs.

Two authors (S.G.V. and G.S.) independently extracted data from articles about study characteristics, which included populations, methods, and results/outcomes, using a prepiloted standard form in order to ensure consistency. Any discrepancies were identified and resolved through discussion (with a third external collaborator where necessary). Due to the nature of the findings, we opted for a narrative synthesis of the results.

## 3. Results

**Figure 1:**
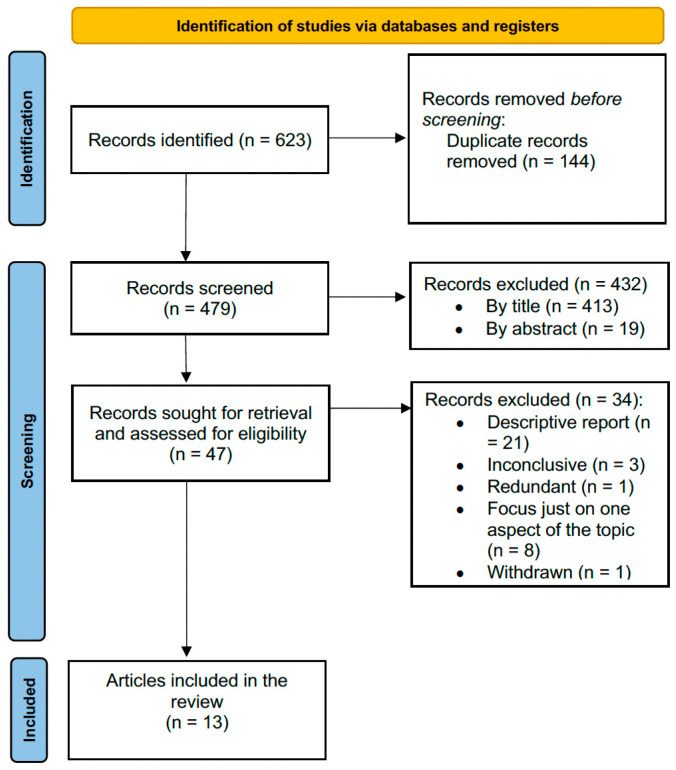
Flowchart for article screening and selection.

Using the reported search strategy, we identified 623 items. After the exclusion of 144 duplicates, we screened 479 items and further excluded 432 of them. The remaining 47 items were selected, and each full text was carefully evaluated in order to select only relevant information (adenomyosis and uterine anomalies). During this process, 33 full texts were considered out of purpose, and 1 was withdrawn; thus, we included the remaining 14 papers that met the abovementioned inclusion criteria (Figure 1).

As summarized in Table 1, ten included studies were case reports [16,17,18,19,20,21,22,23,24,25], one was a retrospective analysis [26], one was a theoretical article [27], and one was a retrospective review [28].

## 4. Discussion

Several congenital syndromes and uterine defects listed mainly in the case reports are shown to be concurrent with adenomyosis, and they may play a role in its development. Indeed, obstructions at the level of the uterus in congenital anomalies may increase intrauterine pressure and promote the penetration of endometrial cells within the myometrial layer and the growth of adenomyotic foci. Nevertheless, adenomyosis can also present as a primary lesion or a secondary one to causes other than an obstruction in syndromic and nonsyndromic CUAs. Mayer–Rokitansky–Küster–Hauser (MRKH) syndrome is a rare congenital disorder with polygenic association characterized by a defect in the development of the paramesonephric (Müllerian) ducts leading to congenital aplasia of the upper two thirds of the vagina, usually replaced by a fibrous septum that joins the rectum with the bladder and to the presence in the place of the uterus of two atrophic hemiuteri with two partial or complete fallopian tubes. As it is Müllerian agenesis, the ovaries have a normal structure and function. The majority of women affected by this pathology present normal secondary characteristics and external genitalia, but they suffer from amenorrhea as well as aplasia of the uterus and upper third of the vagina. Some women, however, may have rudimentary uteri that may contain functioning endometrial tissue, resulting in hematometra, adenomyosis, and pelvic pain. In a study conducted by Hall-Craggs et al. [28], out of 61 patients with MRKH syndrome and nonfunctioning rudimentary uteri identified by magnetic resonance imaging (MRI) of the pelvis, two showed signs of adenomyosis and cyclic pelvic pain. Several genes have been implicated in MRKH syndrome, such as HOXA7, HOXA9–13, HOXD9–13, and WNT4 [29]. These HOX genes are highly conserved genes playing a crucial role in the development of the female reproductive system that relies on a pattern of differential HOX gene expression in the Müllerian duct [30]. Alterations in HOXA expression have been associated with adenomyosis [27], and this may explain its potential relationship with MRKH syndrome, as adenomyosis is not exclusively a secondary lesion in congenital anomalies but can also develop primarily due to (epi)genetic variations [31]. Adenomyosis is also affected by the circulating levels of estrogen. The tissue grows and regresses in an estrogen-dependent fashion, similar to endometriosis [32,33], and polymorphisms in the estrogen receptor alpha gene are associated with a risk of developing adenomyosis [34]. As the ovaries are normal in MRKH syndrome, estrogen-dependent anomalies, such as adenomyosis, may develop.

Other case reports have also suggested a relationship between MRKH syndrome and adenomyosis. In a case reported by Yan and Mok, a 52-year-old woman presented with recurrent lower abdominal pain and fever, and the patient was previously misdiagnosed with primary amenorrhea. The patient was later diagnosed with MRKH syndrome after imaging studies found cervical agenesis, vaginal hypoplasia, and two rudimentary hemiuteri. Moreover, histopathology revealed the presence of adenomyosis in the right hemiuterus. This finding was explained by the unusual development of the uterus in patients with MRKH syndrome, which allowed the misplacement of endometrial cells within the myometrial layer [22]. Another case report presented by Enatsu et al. [23] also reported adenomyosis in a patient with MRKH syndrome. The patient was a 27-year-old Japanese woman that presented with left abdominal cyclic pain. The diagnosis of MRKH was confirmed using laparoscopy, and uterine rudimentary horns were reported bilaterally with normal tubes and ovaries. Moreover, a nodule was found in the left lower abdomen, and histological examinations revealed it was adenomyosis. The suggested hypothesis was that the adenomyosis lesion was a result of metaplasia in the myometrium of Müllerian remnants. In these two cases, the development of adenomyosis in one rudimentary hemiuterus rather than in both suggests that the process is not only due to obstruction, but other etiologies may occur as well.

Other congenital syndromes have also been diagnosed concurrently with adenomyosis. Herlyn–Werner–Wunderlich (HWW) syndrome is a rare congenital urogenital anomaly secondary to mesonephric duct-induced Müllerian anomalies, characterized by uterus didelphys with blind hemivagina and ipsilateral renal agenesis. It usually presents after menarche with progressive pelvic pain during menses, secondary to a palpable mass of increasing volume in the pelvic cavity due to hematocolpos or hematocolpometra [35]. In a study conducted by Zhang et al. [26], among 19 patients studied with HWW syndrome, 1 patient had a presentation complicated with adenomyosis, but the relationship seems unclear. From a speculative perspective, the development of adenomyosis in this rare syndrome may be less likely to occur than in other syndromes as the obstruction is at the level of the vagina; indeed, the vagina can extend and accommodate most of the pressure due to hematocolpos, potentially decreasing the intrauterine pressure and thus the risk of penetration of endometrial cells within the myometrial layer. Another case presented by Ferrero and Bentivoglio [21] reported adenomyosis in a 31-year-old woman with mosaic Turner syndrome (45,X/46,XX/47,XXX) presenting with hypermenorrhea and persistent lower abdominal pain for more than six months. At an exploratory laparotomy, a nodule arising from the posterior uterine wall was noted and removed. Histological examination confirmed the mass as a leiomyoma with focal adenomyosis. As previously highlighted, adenomyosis is an estrogen-dependent lesion, and it may have developed in this patient with Turner syndrome who received estrogen–progestin treatment.

Bicornuate uteri have also been reported concurrent with adenomyosis. Su et al. [19] reported a case of a 41-year-old (gravida 1, para 1) patient with a bicornuate uterus presenting with menorrhagia and dysmenorrhea; the transvaginal sonography revealed a diffuse, heterogeneous myometrium thickening in the left uterine cavity, and pathological assessment of the left myometrium showed adenomyosis. In this patient, pregnancy, interrupting the endometrial basal layer and the myometrium in the left uterus in which it occurred, may have contributed to the development of adenomyosis. Unicornuate uteri have also been reported concurrent with the development of adenomyosis in two cases reported by Frontino et al. [16]. In the first case, an 11-year-old girl with severe dysmenorrhea underwent a diagnostic hysteroscopy, during which a single and regular left tubal ostium was seen, as well as a regular single cervical canal and uterine cavity, resembling that of a left unicornuate uterus. A capsulated inhomogeneous nodule was found on the right side of the uterine fundus as well. Complete resection of the right uterine nodule, along with an ipsilateral salpingectomy, was performed. The nodule was then sliced in the middle, revealing the presence of a small endometrial-like cavity with a hematometra surrounded by a trabeculated adenomyotic-like myometrium, which was confirmed by a histological study. In the second case, a 16-year-old girl presented with acute and severe postmenstrual pelvic discomfort. A single and regular left tubal ostium was seen during diagnostic hysteroscopy, as well as a regular single cervical canal, a uterine cavity similar to that of a left unicornuate uterus, and a 55 × 50 mm^2^ right hypoechogenic nodule. The nodule was split open, revealing a small endometrial-like chamber with a hematometra surrounded by a trabeculated adenomyotic-like myometrium, which was validated by a histologic examination. Feghali et al. [24] also reported the case of a left unicornuate uterus with a right-sided noncommunicating rudimentary horn diagnosed using MRI. The horn was laparoscopically removed and sent for pathological studies to reveal the presence of diffuse adenomyosis. The latter mainly develops due to the obstructive consequences of the horn on the active endometrium leading to the distention of the cavity and sometimes adenomyosis. Similarly, Morelli et al. [25] reported a case of coexisting adenomyosis with an extremely distant noncommunicating uterine horn and myoma, treated with a laparoscopic hemi-hysterectomy. In addition, for this case, the diagnosis of adenomyosis was available only in the final histological report.

Finally, Kim et al. reported a case of a 38-year-old woman (gravida 0, para 0) with severe menorrhagia and dysmenorrhea. A uterus didelphys was confirmed using MRI imaging of the pelvis, which revealed two totally separated uteri and two cervices. On a T2-weighted scan, the JZ of the left uterus was 40 mm thick, with punctate, high-signal-intensity myometrial foci, suggesting localized adenomyosis [17].

To the best of our knowledge, this is the first review specifically focused on reporting the concurrent diagnosis of adenomyosis and CUAs. Nevertheless, the available pieces of evidence were retrieved mainly from case reports and retrospective cohorts with few patients. In addition, there are also cases where adenomyosis is not observed in the uterus; therefore, the result of this review could not be considered as a basis for relevance. For these reasons, the relationship between adenomyosis and CUAs needs further studies to be fully elucidated.

## 5. Conclusions

Adenomyosis can be found in both syndromic and nonsyndromic CUAs and may arise from several etiologies. The hypothesis that obstructions in CUAs increase uterine pressure and promote the development of adenomyosis remains to be further elucidated, and additional findings may also play a role. The patient’s genetic, epigenetic, and hormonal patterns, as well as normal physiological processes, such as pregnancy, may influence the growth of adenomyosis. Future studies are needed to evaluate whether a potential cause–effect mechanism may occur in cases with such concurrent diagnosis.

## Figures and Tables

**Table 1:** Characteristics of the included studies.

Author	Year	Type	Congenital Abnormality	Country	Patient (n)	Age (Mean)
Frontino et al. [16]	2009	Case report	Unicornuate uterus	Italy	2	13
Kim et al. [17]	2011	Case report	Uterus didelphys	Korea	1	38
Takeuchi et al. [18]	2003	Case report	Septate uterus	Japan	1	56
Zhang et al. [26]	2019	Retrospective analysis	HWWS syndrome	China	1	17.67
Du et al. [27]	2015	Theoretical article	-	US	-	-
Hall-Craggs et al. [28]	2013	Retrospective review	Rudimentary uteri in MRKH syndrome	UK	2	19
Su et al. [19]	2005	Case report	Unicornuate uterus	Taiwan	1	41
Narayanan et al. [20]	2015	Case report	Müllerian remnants in MRKH syndrome	India	1	43
Ferrero and Bentivoglio [21]	2004	Case report	Mosaic Turner syndrome	Italy	1	31
Yan and Mok [22]	2002	Case report	Rudimentary uteri, cervical agenesis, and vaginal hypoplasia in MRKH syndrome	Hong Kong	1	52
Enatsu et al. [23]	2000	Case report	Rudimentary uteri in MRKH syndrome	Japan	1	27
Morelli et al. [25]	2013	Case report	Noncommunicating uterine horn	Italy	1	41
Feghali et al. [24]	2022	Case report	Unicornuate uterus	Lebanon	1	20

MRKH: Mayer–Rokitansky–Küster–Hauser syndrome. HWWS: Herlyn–Werner–Wunderlich syndrome.

## Data Availability

Full data extraction will be available from the last author (Z.K.) on reasonable request.
